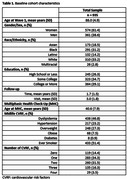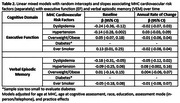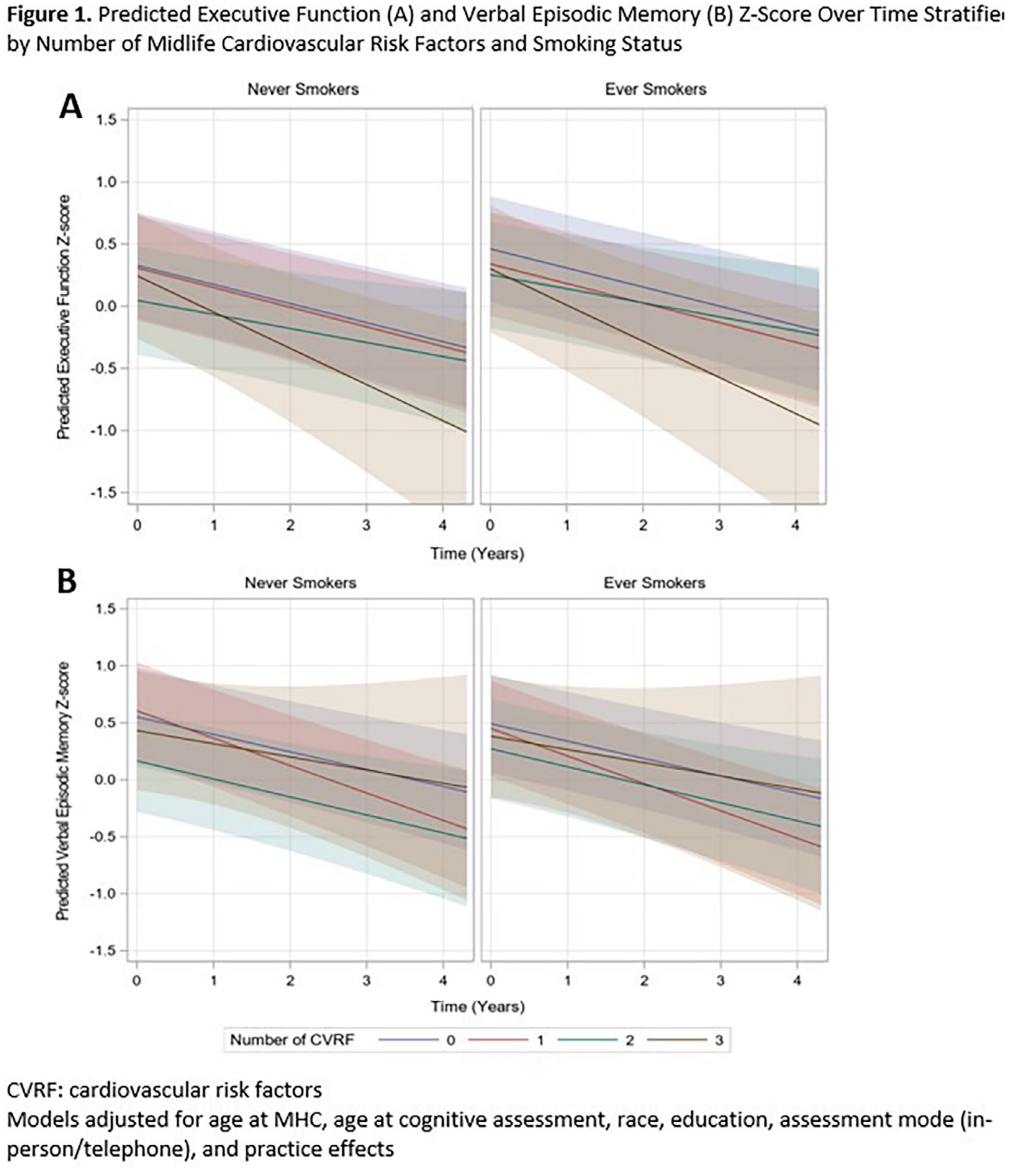# Midlife Cardiovascular Risk Factors and Cognitive Decline in a Diverse Cohort of Adults Ages 80 and Older

**DOI:** 10.1002/alz.092785

**Published:** 2025-01-09

**Authors:** Kristen M. George, Paola Gilsanz, María M. M. Corrada, Alexander Ivan B. Posis, Rachel Peterson, Lisa L. Barnes, Claudia H. Kawas, Dan M. Mungas, Charles Decarli, Rachel A. Whitmer

**Affiliations:** ^1^ University of California, Davis, Davis, CA USA; ^2^ Kaiser Permanente Northern California Division of Research, Oakland, CA USA; ^3^ University of California, Irvine, Irvine, CA USA; ^4^ University of Montana, Missoula, MT USA; ^5^ Rush University Medical Center, Chicago, IL USA; ^6^ University of California, Davis, Sacramento, CA USA

## Abstract

**Background:**

Midlife cardiovascular risk factors (CVRF) are associated with late‐life cognitive impairment, but also mortality. We evaluated their association with cognitive decline in a diverse cohort of older adults who survived to age 80 without diagnosis of dementia.

**Method:**

Three harmonized cohorts of long‐term Kaiser Permanente members were pooled and restricted to those aged 80+. We measured CVRF from Multiphasic Health Check‐ups (MHC; 1964‐1985), which assessed hyperlipidemia, hypertension, overweight/obesity, diabetes, and smoking status. Standardized z‐scores for executive function (EF) and verbal episodic memory (VEM) were measured over up to 8 waves (2017‐2023) using the Spanish and English Neuropsychological Assessment Scales. Linear mixed models with random intercepts and slopes associated CVRF, separately, with cognitive decline adjusting for age at MHC, age at cognitive assessment, race (Asian/Black/Latino/white/multiracial), education (≤high school/some college/≤college), assessment mode (in‐person/telephone), and practice effects. Sensitivity analyses evaluated the count of CVD risk factors stratified by smoking status.

**Result:**

Participants (n = 935) averaged 40.6(±7.9) years of age at MHC, 88.0(±4.9) years at baseline cognitive assessment, and 61.4% were women (Table 1). At midlife, 47% had hyperlipidemia, 23% hypertension, 34% overweight/obese, 1% diabetes, and 51% ever smoked. Hyperlipidemia was associated with worse baseline EF (β(95% CI):‐0.24(‐0.36,‐0.12)), smoking was associated with better EF (β:0.13(0.01,0.25)), and hypertension was associated with worse EF (β:‐0.14(‐0.25, 0.002)), though the effect was not statistically significant (Table 2). There were no associations between CVRF and EF decline. Hyperlipidemia was the only CVRF associated with worse baseline VEM (β:‐0.18(‐0.31,‐0.05)), and no CVRF was associated with VEM decline (Table 2). Despite smokers having higher baseline EF compared to nonsmokers, having 3+ CVRF was associated with steeper decline in both groups compared to those with fewer CVRF (Figure 1). Baseline VEM was similar between smokers and non‐smokers, and for both smokers and nonsmokers, the steepest declines in VEM were among those with one CVRF.

**Conclusion:**

In this diverse cohort, midlife hyperlipidemia was associated with worse cognition in late life. A positive association between smoking and EF may be indicative of resilience among midlife smokers who have reached age 80+. Regardless of smoking status, multiple CVD risk factors may be detrimental to cognitive function, particularly EF.